# Healthcare Providers’ and Frontline Workers’ Experiences of an Ebola Vaccine Trial in the Boende Health District of the Democratic Republic of the Congo

**DOI:** 10.4269/ajtmh.23-0581

**Published:** 2024-07-02

**Authors:** Trésor Zola Matuvanga, Freddy Bikioli Bolombo, Antea Paviotti, Ynke Larivière, Gwen Lemey, Maha Salloum, Bernard Isekah Osang’ir, Junior Matangila, Vivi Maketa, Emmanuel Esanga, Solange Milolo, Patrick Mitashi, Pierre Van Damme, Hypolite Muhindo-Mavoko, Jean-Pierre Van Geertruyden

**Affiliations:** ^1^ Centre for the Evaluation of Vaccination, Vaccine and Infectious Disease Institute, University of Antwerp, Wilrijk, Belgium;; ^2^ Global Health Institute, Department of Family Medicine and Population Health, University of Antwerp, Wilrijk, Belgium;; ^3^ Tropical Medicine Department, University of Kinshasa, Kinshasa, Democratic Republic of the Congo;; ^4^ Division Provinciale de la Santé de la Tshuapa, Ministry of Health of the Democratic Republic of the Congo, Kinshasa, Democratic Republic of the Congo

## Abstract

This study explored the experiences of healthcare providers (HCPs) and frontline workers who were involved in an Ebola vaccine trial in the Democratic Republic of the Congo. The researchers interviewed a total of 99 participants (HCPs and frontline workers) living and working in the Boende health district during the period of the study, from February to March 2022. These individuals included a mix of trial participants and non-trial participants (staff of the trial, local health authorities, and head nurses of health centers). In-depth individual interviews, as well as focus group discussions (FGDs), were used to understand interviewees’ experiences and perceptions. The data were analyzed to identify the main themes. The findings unveiled a multitude of positive experiences among interviewees/FGD participants. The commitment of the trial investigators to improve the study site and to equip the volunteers with necessary skills and knowledge greatly contributed to a positive trial experience. However, some interviewees felt that the reimbursement for time and travel expenses during their trial visits was insufficient in comparison with their expectations. Additionally, there were expressions of worry about the frequency of blood draws during scheduled trial visits. Our findings emphasize the critical importance of addressing and continuously considering the perspectives and concerns of trial participants before designing and implementing vaccine trials. By actively incorporating their inputs, researchers can mitigate concerns and tailor communication strategies, potentially enhancing the overall success and impact of the vaccine trial.

## INTRODUCTION

Ebola virus disease (EVD) has a recurrent presence in the Democratic Republic of the Congo (DRC), and there remains a risk of reemergence from an animal reservoir or through relapse among survivors.^1^^–^^3^ The rise in the occurrence of EVD outbreaks observed from 2020 to 2023 in the DRC is rooted in previous outbreaks due to relapses among survivors.^3^^,^^4^ At least one outbreak has occurred each year from 2017 to 2022 in both the Equateur and North Kivu provinces in the DRC.

Prophylactic vaccination against EVD is not yet part of the standard prevention strategy (at the time of writing this article). However, the proximity of successive outbreaks in recent years in the same regions of the DRC, particularly in remote areas, underscores the need to continue efforts to provide prophylactic vaccination to specific at-risk populations, including health care providers (HCPs) and frontline workers. These individuals are at a much greater risk due to their frequent and close contact with patients who may be infected with the virus and can act as amplifiers of transmission during outbreaks.^5^ It is worth highlighting that even minimal deaths among HCP populations in a remote area of the DRC can have disastrous effects on a health care system already weakened by several endemic diseases, including malaria, and the recurrence of epidemics such as measles and monkeypox, adding strain to an already fragile health care system.^6^ Prophylactic vaccination of HCPs and frontline workers before the start of an EVD epidemic has great potential to significantly reduce the number of EVD cases and death rates and to mitigate its impact on the health system.^6^^,^^7^

In 2014, the Boende health district experienced an outbreak resulting in 66 cases and 49 deaths. Furthermore, in 2022, the initial case of the 14th EVD outbreak in Mbandaka, located in the neighboring Equateur province, was traced back to an individual who had recently returned from a vacation in the Boende health district, a month prior to experiencing symptoms. Notably, this individual was a medical student who had recently concluded his vacation internship at the general referral hospital of Boende. No contact with an EVD survivor was reported. Given the persistent zoonotic exposure in this region, it is challenging to accurately forecast the occurrence of an epidemic, implying that the risk of such exposures may endure indefinitely.

Within the framework of the European Union’s EBOVAC3 project (IMI-EU), the University of Antwerp and the University of Kinshasa conducted an Ebola vaccine trial known as EBL2007 (NCT04186000). The EBL2007 vaccine trial, an open-label, randomized, phase 2 study, evaluated the immunogenicity and safety of the Ad26.ZEBOV and MVA-BN-Filo vaccine regimen and an Ad26.ZEBOV booster dose in 699 registered HCPs and frontline workers (e.g., medical doctors, nurses, midwives, community health care workers, first aid workers, laboratory technicians, health facility cleaners, hygienists, caregivers, pharmacist aids, nutritionists, and vaccination program aides) between 2019 and 2022. We considered HCPs as those who work in a health facility and might come into contact with infected patients in this facility (e.g., doctors, nurses, midwives, laboratory technicians, and health facility cleaners, etc.) and frontline workers to be those with a profession leading to early potential Ebola virus (EBOV) exposure in the community (e.g., first aid workers, community health care workers, stretcher bearers, and caregivers, etc.).^8^ It is crucial to highlight the following aspects concerning volunteering for the EBL2007 vaccine trial. First, to enhance potential participants’ understanding of the trial, a test of understanding (TOU) was carried out after the study protocol had been explained. The TOU consisted of a pretested, structured questionnaire (comprising closed-ended questions) devised by the EBL2007 trial investigators to evaluate the potential participant’s grasp of the study’s essential information and requirements. This TOU was conducted prior to the signing of the consent form during the screening visit. Successful completion (≥9/10; three attempts possible) was a prerequisite for signing the informed consent and an inclusion criterium for enrollment the trial. Second, regarding reimbursement for time and travel expenses, participants who traveled for less than 6 hours were provided with a fixed amount of $20 to cover transportation costs, while those who traveled for more than 6 hours received a reimbursement of $40, which was allocated to cover expenses associated with food and accommodation.^9^

Although the Ad26.ZEBOV–MVA-BN-Filo vaccine regimen has been approved by the European Commission for the prevention of EVD since July 2020, the findings of this study are anticipated to generate supplementary data from a new population, thereby contributing to a more comprehensive understanding of this vaccine regimen.^10^ Numerous challenges were encountered pertaining to the issue of mistrust surrounding a different Ebola vaccine (the rVSV-ZEBOV vaccine) during another vaccine trial conducted amidst the 10th EVD epidemic in the eastern provinces of the DRC (North Kivu and Ituri).^11^^,^^12^ Some Ebola patients and Ebola contacts (suspected cases) actively rejected vaccination and visits to the proposed EVD treatment center because they did not believe in the existence of the Ebola virus.^13^^–^^15^ This contributed to a prolonged epidemic, the most widespread and long-lasting in the recorded history of EVD to this day in the DRC (2018–2020). Additionally, results from other studies on the acceptance of Ebola vaccines among HCPs suggest that it is not easy to predict the acceptability and perception of a nonapproved vaccine.^11^^,^^14^ Being aware of these forms of hesitancy and resistance, we carried out a qualitative investigation during the follow-up period of the EBL2007 trial to understand how it was perceived by participants, trial staff, and health authorities.

## MATERIALS AND METHODS

This qualitative study was nested within the above-mentioned vaccine trial conducted in the Boende health district, a remote area in the DRC.^8^

### Study setting.

**Figure 1.**
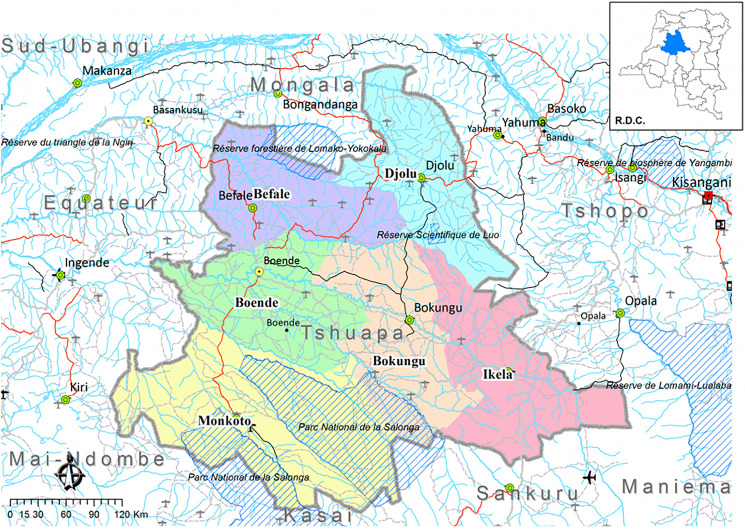
Tshuapa and the health district limits.^18^

The Boende health district, one of 12 health districts in the Tshuapa province in the DRC encompassing six territories and the capital city of Boende (Figure 1), is home to about 296,253 residents.^16^ This district is characterized by the presence of two ethnic groups (Bantu and Pygmies), with the Bantu ethnic group being predominant. The languages mostly spoken across the province are Lomongo, Lingala, Longando, Topoke, and French, with French being used in administration and education. The most practiced religions include Catholicism (with over half of the population in the DRC identifying as Roman Catholic) and minorities practicing Protestantism, Kimbanguism, Islam, Revivalism, Brahmanism, Jehovah’s Witnesses, Banga Nzambe, and Kitawalism, etc.^17^ In spite of that, traditional religious beliefs heavily influence local beliefs and practices.^18^

The main way of traveling to other cities for the local people is on an improvised boat, commonly called a whaleboat (Supplemental Appendix 1), which poses a frequent risk of crashes and overturning, resulting in the loss of human lives, as well as motorcycles and biking. Traveling by plane is also possible but not a common mode of transportation for most residents.

The provision of health care services within the province is ensured through governmental structures such as health centers (centre de santé), referral health centers (centre de santé de référence), and general referral hospital (hôpital général de référence), as well as other private facilities; health care services are also offered by nongovernmental organizations and religious organizations. Traditional medicine represents a significant portion of the health system, as visits to traditional healers represent a significant portion of health-seeking behavior, either due to the financial cost associated with seeking treatment in health facilities or for treating specific conditions such as fractures and mental disorders, etc., which are not considered curable or can be only partially cured by the modern medicine available in this remote location.

### Participants’ recruitment and sampling.

A purposeful sampling technique was used, and interviewees were invited per their role in the EBL2007 vaccine trial, gender, and health care work categories. Facilitated by the EBL2007 vaccine trial site coordinator, the researchers extended invitations to a total of 117 HCPs and frontline workers who either participated or discontinued participation in, or did not participate in, the EBL2007 vaccine trial, and local health authorities of the province of Tshuapa.

From a literature review,^8^^,^^9^^,^^19^^–^^22^ methodological tools were developed and included a topic guide, outlining key issues and serving as a basis for designing a discussion framework for the focus groups and in-depth interviews (Supplemental Appendix, Supplements 1–4).

Perceptions related to the EBL2007 clinical trial were discussed with trial participants, those withdrawn from the trial, and nonparticipants (trial staff, local health authorities, and head nurses of health centers). These perceptions included, more specifically, their experiences and perspectives on the services rendered and/or obtained throughout the EBL2007 vaccine trial, the challenges faced by trial staff/trial participants and how they were overcome, internal and external collaboration and communication (between trial staff and trial participants and between trial staff and local health authorities), the acceptability of the study vaccine, the motivation for participating in the trial, and the expectations and hopes for the future of the study site and Ebola vaccine research.

### Data collection methods.

Study data were collected during focus group discussions (FGDs) and in-depth individual interviews (or one-on-one interviews), using adapted, open-ended, pretested interview guides (Supplemental Appendix, Supplement 2).

### Focus group discussion.

To gather firsthand accounts from HCP and frontline worker participants in the EBL2007 vaccine trial, FGDs were conducted. For the FGDs, trial participants were grouped according to their sex and occupation. In each FGD, 6 to 10 individuals participated. One FGD assembled registered head nurses of health centers (infirmiers titulaires) that were not part of the trial. All the FGDs followed the same interview guide, which ensured a systematic approach to data collection (Supplemental Appendix, Supplement 1). The questions in the guide were pretested, reviewed, and refined during the fieldwork based on emerging themes from the discussions. The FGDs encompassed the following themes: general understanding of the EBL2007 vaccine trial, motives behind the decision to participate, experiences as participants throughout the trial and its finalization, expectations and hopes for the trial, perceptions of the trial’s impact on participants’ daily lives and the community, and perspectives on the future of the trial site and Ebola vaccine research after their participation. Discussions were conducted in either French or Lingala, depending on the group’s preference.

### Individual in-depth interviews.

The interviews used a semistructured grid (Supplemental Appendix, Supplements 2 and 3) to capture testimonies regarding the EBL2007 vaccine trial. The following areas were covered: difficulties encountered and strategies for overcoming them, psychological well-being during the trial, collaboration and communication with other medical staff and stakeholders, perceptions of the vaccine trial in Boende and relationships with the community, perspectives on the future of the trial site and capacity building received during the trial, and prospects for career trajectory after the trial concludes.

The narratives of participants who withdrew consent encompassed several key aspects, including their overall comprehension of vaccine trials and specifically the EBL2007 trial protocol in Boende. Additionally, they shared insights into the factors that influenced their decision to withdraw consent.

Insights from HCP and frontline worker participants in the trial or staff, as well as local authorities such as the provincial minister of health, the chief of the provincial health division (Chef de la Division Provinciale de la Santé) of Tshuapa, and the head of the Boende health district were captured using a pretested semistructured questionnaire (Supplemental Appendix, Supplement 4).

### Procedures.

Prior to the start of an interview (FGD or individual in-depth interviews), participants were reminded of the purpose of the study. Each conversation lasted 60 to 90 minutes, and participants’ statements were audiotaped. The collected voice recordings in a language other than French were translated into French prior to being transcribed by two independent transcribers not involved in the trial. The correctness and consistency of transcripts were double-checked by T. Zola Matuvanga; he coded all transcripts obtained from all FGDs and in-depth interviews. A. Paviotti and T. Zola Matuvanga agreed on the codes and categories used to create themes.

The FGDs were facilitated by a postdoctoral researcher (A. Paviotti), who sometimes acted as the moderator or note-taker, while two doctoral students (T. Zola Matuvanga and F. B. Bolombo) also intermittently acted as note-takers or moderators. One of the doctoral students, T. Zola Matuvanga, also acted as a subinvestigator of the EBL2007 vaccine trial, while F. B. Bolombo was not involved in the trial activities.

All individual in-depth interviews were conducted by T. Zola Matuvanga, A. Paviotti, or F. B. Bolombo in a carefully selected and tranquil setting preferred by the interviewees. Each interview had an average duration of approximately 50 minutes, allowing for in-depth exploration of the topics discussed.

## ANALYSIS

The analysis was conducted using a thematic approach, employing both inductive and deductive coding strategies. Recordings of discussions and interviews were transcribed and, if necessary, translated into French. The transcribed data were then imported into NVIVO software for analysis. Each transcript was anonymized and attributed a unique identifier. Using the NVIVO software, A. Paviotti and T. Zola Matuvanga held regular meetings to harmonize the coding. Initial codes were derived from predefined themes in the interview guide. These codes were grouped under the predetermined themes using a starting list. Subsequently, an inductive approach was adopted, enabling the identification of new codes that were not initially anticipated. The starting list was updated to incorporate emergent codes. An iterative process was employed to further refine and develop the sub-themes and themes, ensuring a coding process in line with the objective.

### Ethical considerations.

The National Ethics Committee of the DRC Ministry of Health gave its approval before the study commenced (Avis no. 368/CNES/BN/PMMF/2022). At the start of each discussion, it was made clear to all potential participants that their involvement was optional and voluntary. Informed consent was given verbally by all those participating (in French or Lingala depending on the participant’s preference) before the interviews or FGDs.

## RESULTS

A total of 10 FGDs and 15 in-depth interviews were conducted. In total, 99 HCP and frontline worker EBL2007 trial participants, non-trial participants, people withdrawn from the trial, and local health authorities responded favorably to partake in this study, as described in Table 1. Data saturation was reached after FGDs and in-depth individual interviews in a sample of 99 willing respondents were conducted. Respondents in the FGDs and in-depth interviews conducted were 53.5% male (53/99). The mean age of the interviewees and FGD participants was 52 (±10.9) years, ranging from 28 to 69 years.

**Table 1:** Interviewees by data collection methods and characteristics

Relation to the EBL2007 vaccine trial (participant category)	Occupation	Data collection method	Male (*n*)	Female (*n*)	Total (*N*)
Trial participants	Nurse	2 FGDs	10	8	18
	First aid worker	2 FGDs	9	8	17
	Community health worker	2 FGDs	8	7	15
	Midwife	1 FGD	–	9	9
	Health facility cleaner	1 FGD	2	8	10
	Medical doctor	1 FGD	6	–	6
Non-trial participants	Head nurse of health centers (Center de Santé)	1 FGD	10	–	10
Total		10 FDGs	45	40	85
Staff in the trial	Laboratory technician	2 In-depth individual interviews	1	1	2
	Nurse	2 In-depth individual interviews	1	1	2
	Sentinel (watchman)	1 In-depth individual interview	1	–	1
	Cleaning lady	1 In-depth individual interview	–	1	1
Withdrawn from trial	Anonymous	4 In-depth individual interviews	2	2	4
Local health authority	Provincial minister of health	1 In-depth individual interview	–	1	1
	Head of the health division of Tshuapa	1 In-depth individual interview	1	–	1
	Chief of the health district	1 In-depth individual interview	1	–	1
	Directorate of the general referral hospital of Boende	1 In-depth individual interview	1	–	1
Total		15 In-depth individual interviews	8	6	14

FGD = focus group discussion.

Prior to presenting our research findings, it is crucial to provide a contextual framework to better understand the responses provided by the interviewees. The recruitment phase of the EBL2007 vaccine trial commenced in December 2019, marked as the day 1 visit, and the vaccine regimen under investigation had not yet received approval for use in the European Union (EU) at that time. Study participants received the study vaccines at 56-day intervals between December 2019 and April 2020. During the initial visit, participants were randomized into two groups, with one group scheduled to receive a booster vaccination after 1 year (2020–2021) and the other group scheduled to receive a booster vaccination after 2 years (2021–2022) after the day 1 visit. Consequently, the year 2 visit served as the final scheduled visit for the first group, while the second group was expected to continue with safety follow-ups for 6 more months.

Our research was conducted shortly after the year 2 visit, coinciding with the completion of all visits for the first group. It is worth noting that the recruitment and follow-up period of this trial coincided with the occurrence of five EVD outbreaks in the provinces of Equateur and North Kivu (see Supplemental Appendix 2, Table 1), and it is important to highlight that no EVD epidemic was officially declared in Boende during the entire duration of the trial activities.

Upon analysis of the data, the experiences of both participating and nonparticipating HCPs and frontline workers, trial staff, and local health authorities were categorized into three major themes: overall perceptions of the trial experience, appreciations of the trial, and perspectives on future Ebola/Ebola vaccine research. The themes, including identified subthemes and categories, are described below.

### Overall perceptions of the trial.

#### Aims of the trial.

The objective of the EBL2007 vaccine trial was perceived by some interviewees and FGD participants as an initiative/project to assess whether the investigational vaccine confers protection and to describe any adverse reactions associated with vaccination during the trial’s inclusion period. However, the participants correctly recalled that receiving the vaccine did not necessarily mean that they would be protected against Ebola or that they could be exposed to Ebola risk practices without contracting Ebola:

“The primary objective of our research was not centered around personal protection against Ebola virus disease*, *but only to study it. The vaccine was not brought to protect someone so that someone may say: ‘not as I am vaccinated with EBOVAC …. I see someone who has the Ebola virus who must bite me because I am protected’…*. *It doesn’t mean that we are sure that we are protected or immune ….” (FGD, nurse participant in the trial, woman).

“[…] We observed all these people who received the vaccine, and that’s the objective, to see how people will react to the Ebola immunization during a certain period.” (Interview, local health authority, man).

#### Achievement of objectives in terms of expectations.

Some respondents expressed a mix of frustration, anger, and sadness as the trial neared its end. They felt that this ending was unexpected, particularly because the promised results (which were not yet available when data were being collected) had not yet been shared with them. These participants believed that disclosure of the vaccine study results or feedback on the collected blood samples would be perceived to acknowledge and value their altruistic contributions to the science.

“Well! What they said: ‘We took the samples’ and then they left. As such Gwen^1^ had said if it is after 6 months that they are going to come back with the results[…] So, on that point I don’t know if it will be like that.” (Interview, trial staff, nurse, woman).

“[…] given the experience of Lokolia,^2^ we told ourselves: ‘even if we are taken as guinea pigs, we may sacrifice ourselves for the others’, but otherwise we were waiting, until today, we are waiting for the results of the study that was conducted here […]” (FGD, trial participant, medical doctor, man).

However, the conclusion of the trial was generally regarded as a major triumph by the trial staff, as it marked the culmination of a 2.5-year endeavor in a remote area of the DRC. Notably, the study demonstrated an impressive retention rate of over 90%, further highlighting the success and dedication of the teams and participants involved. Several interviewees emphasized that the objectives of the trial exceeded their initial expectations. This sentiment arose from the recognition that, alongside the trial vaccine, additional components were incorporated, such as training of HCPs and frontline workers volunteering in the trial, from which the area of Boende would inevitably benefit by improving health care delivery.

“[…] Although we initiated the study with a considerable number of participants, it is important to acknowledge that a small proportion was lost to follow-up. However, it is noteworthy that most of these losses did not significantly impact the overall participant cohort. The study commenced with an initial cohort of 700 participants. However, the attrition rate remained relatively low, as we did not experience a loss of more than 100 participants […] I don’t have the count on my head, but we have about 600 and some participants like that.” (Interview, medical doctor, trial staff, man).

“[…] So in the study, even if it was not stated in the protocol[…] but other things we did with the participants, it strengthened their capacity to deliver and even in what they do, especially the first aid worker, they had a lot of training sessions, and they were very happy that they themselves were starting to train their participants.” (Interview, local health authority, man).

A few respondents expressed concern regarding the large portion of the population that was not part of the project, which they expected would be expanded in the province and to the entire population, as in the following statements:

“In my opinion, the goal is achieved for some, but not for others. You did the injustice, why you called some and you left others?” (Interview, health facility cleaner, trial staff, woman.)

“When a disease emerges, it does not discriminate based on occupation or whether one works in the healthcare sector. The disease affects the entire community. Personally, I strongly advocate for larger vaccination among all community members.” (Interview, trial participant who withdrew consent, man).

#### Informed consent.

A significant number of interviewees and FGD participants perceived volunteering in the EBL2007 vaccine trial as establishment of a contractual relationship with the research team. This was to result in a long-term plan to address any harm related to the experimental product. Given the contractual perception of the study consent document among trial participants, some of them expected consistent financial benefits in relation to the risk and time spent in the trial accordingly. This concern caused some to leave the study during the follow-up:

“My question was the following; a community healthcare worker asked me: ‘[…] Were we paid in the risk that we had taken of testing this vaccine in our body?’ I told them, I also want to ask the study coordinator. The community healthcare workers are going to go home empty-handed?” (FGD, community health care worker, trial participant, man).

Some of the interviewees who withdrew from the trial expressed the opinion that the expected money offered for travel costs and time spent in the trial was deemed insignificant.

“I believed that I would have consistent payment for travel cost and time offered when entering this trial. That is what motivated me to enter, but it was insignificant. That’s what made me feel discouraged even if I quit[…] there was not much[…]” (Interview, trial participant who withdrew consent, woman).

“I did not encounter difficulties during the study; everything was going well except for the payment that had discouraged me.” (Interview, trial participant who withdrew consent, woman).”

#### Motivations for discontinuation.

A considerable number of interviewees voiced concerns regarding the frequency of blood withdrawals during each scheduled visit in the trial, as well as safety issues associated with the study vaccine. These concerns ultimately led to their decision to discontinue their participation. As far as blood is concerned, a few participants found it unclear why the collected blood samples were being sent to laboratories located overseas. The blood collection is perceived as not permissible according to certain religious beliefs of some interviewees like Jehovah’s Witnesses, who highlighted that blood should not be distributed or tampered with.

“No, blood is something that is in someone’s organism. My blood is my blood, yours is yours. I may not take my blood away to give you, if I have germs, it will happen to you. Don’t you think it’s bad? Even the Bible doesn’t want it that way[…] We fear God, we don’t fear anyone[…]” (Interview, trial participant who withdrew consent, man)”.

Some health issues after vaccination in the trial were perceived by some respondents as being caused by the investigational vaccine, as indicated in the following statements.

“Upon initiation of the trial, I began experiencing pain, which initially affected my entire body. However, over time, the pain has become localized to my feet. Furthermore, during the time I participated in the trial, I also developed oedema in my lower limbs.” (Interview, trial participant who withdrew consent, woman)

“No, I declined participation solely due to observed changes in my body, but it was not due to any negative experiences.” (Interview, trial participant who withdrew consent, woman).

During the interviews, it became evident that a portion of the respondents had no recollection whatsoever of the information presented to them during the informed consent process, which had taken place 2 years prior. Their memory seemed to have failed regarding the reasons behind their voluntary participation in the vaccine trial for example, as stated by one interviewee:

“I was sitting somewhere[…] Well, we were invited, and I didn’t know the procedures of the study and I didn’t know that they were going to draw blood. The first time I got there I didn’t know, the second time it was the same, when the third time came, my conscience was worked on. I’m a Christian, Jehovah’s Witness, for us the blood[…] They take the blood and put it on you, it’s a sin, and it was the conscience that worked on me, and I made the decision to stop.” (Interview, trial participant who withdrew consent, man).

#### Study vaccine acceptability and motivations.

The firsthand experience of the 2014 EVD epidemic and the prevailing concern regarding a potential outbreak in the foreseeable future played a fundamental role in the widespread acceptance of the study vaccine among interviewees and FGD participants.

“What pushed me to accept, was that the vaccine in question, if we accept it, it will help us for the next epidemic[…] if the virus catches you and if you were vaccinated, the disease severity will not be fatal.” (Interview, trial participant who withdrew consent, woman)”.

Furthermore, the provision of ancillary care (i.e., the care provided to participants that goes beyond the research aims or intervention), along with the reimbursement of travel expenses and offered compensation for the time dedicated to each scheduled visit in the trial, emerged as predominant motivating factors for other respondents.

“I was interested in this study because of the money I was given for transportation[…] It helped me to buy things to eat.” (FGD, nurse, trial participant, woman).

“[…] What pushed me is that I am a community health worker[…] Well the community health worker works on a volunteer basis but they have a little motivation for the […] It depends on the agencies or the NGO, but when EBOVAC came, I decided to join because I heard that there was also the motivation of transportation reimbursement.” (Interview, trial participant who withdrew consent, man).

“When we joined the study, it was good. For instance, if you fell ill, we would support your medical care until you were discharged from the hospital. If you were only hospitalized, we would continue your care until you were discharged. And if you came solely for medical treatment, we would prescribe medication and provide it to you free of charge. It was truly for our benefit.” (FGD, midwife, trial participant, woman)

#### Vaccine safety.

Many interviewees and FGD participants reported safety concerns about the study vaccine as they experienced events such as abortions, onset of diabetes, high blood pressure, back pains, or gastritis, which occurred suddenly after vaccination in the study and were perceived as related to the study vaccine.

Since the study vaccine was not recommended in pregnant women and those intending to become pregnant within 3 months, a few participants questioned the relationship between the study vaccine and pregnancy. The administration of the vaccine was even perceived by a few respondents as facilitating their ability to conceive and become pregnant, while for those who got pregnant after vaccination and experienced pregnancy loss, these miscarriages were perceived as being caused by the vaccine. Those who experienced pregnancy loss expressed this as a concern about the acceptability of this experimental vaccine when the immunization efforts are expanded to the broader community beyond the HCPs and frontline worker population. Nevertheless, the reimbursement of expenses following a medical incident played a role in bolstering the level of confidence among most trial participant respondents.^23^

“Some people say: ‘since when I have received the vaccine, I am too sick.’ Some other people say: ‘since when I have received the vaccine, I am like before.’ Other people say: ‘since when I have received the vaccine, I am fine’[…] There is a mother who told us: ‘I never got pregnant; after receiving the vaccine, now I am pregnant.’ She is very happy, for her, the vaccine has done something for her.” (Interview, nurse, trial staff, woman).

“Some of the trial participants asked me to say this: ‘Doctor, since when I have been in this trial, since I got the vaccine, I noticed that when I conceive, 2 months or 3 months later, I lose conception, menstruation comes back, appears. I wonder if it’s not related to the vaccine.’” (Interview, nurse, trial staff, woman).

#### Acceptability of the study site and target population.

When contemplating the suitability of the study site, most interviewees and FGD participants indicated that the trial should have been conducted in the vicinity of Lokolia and its surrounding health areas, where the previous 2014 Ebola outbreak happened. Additionally, some respondents questioned the representativeness of the HCPs, because those residing in proximity to the 2014 outbreak area were not enlisted or invited for the screening procedures in the study.

“This study should normally be carried out in Lokolia. Where there was an epidemic, where there is the community, the people who lived it. As Lokolia is a health area in the Boende health district, it’s not bad, but the sample[…] You should have had to recruit a lot of people from Lokolia, but in the 700, if you observe, there are only[…] Even the head nurse [of Lokolia] was not involved.” (FGD, nurse head of health center, non-trial participant, man).

### Communication.

#### Interactions between trial participants, nonparticipants, and local authorities.

Some local hospital authorities raised concerns about the employment of some staff of the HGR of Boende, along with the use of some hospital premises. This potentially diverted resources among the HCP staff most involved in the EBL2007 vaccine trial. To reach an agreement, the investigators had to preserve transparent communication indicating that trial conduct would neither interfere with the medical care of patients nor affect the hired hospital staff’s workload. Therefore, in this regard, each HCP used in the trial had to ensure that he/she had a backup so as not to interrupt general care services. Additionally, the number of invited HCP and frontline worker volunteers per visit was limited, per professional categories, in order not to leave some health care services empty. Finally, certain local health authorities expressed a desire to establish permanent training programs for HCPs working in the Boende health district, via a partnership with international sponsors of the trial, to ensure a lasting impact and knowledge transfer in this remote area.

“Indeed, there were instances where interactions with the staff, particularly at the hospital, were challenging, primarily involving the hospital director. Initially, there were concerns as a portion of the department was relocated and merged with other units to accommodate the implementation of the EBOVAC project …” (Interview, local health authority, man).

#### Rumors.

Circulating rumors suggested that the vaccine trial was perceived as an established or prearranged agreement between the research team and the vaccine manufacturer. The alleged purpose of this agreement was either to intentionally cause delayed mortality in the vaccine recipients or to reduce their life expectancy. Consequently, the act of collecting blood samples during scheduled visits in the trial was perceived as a mechanism to seal this alleged deal. These rumors reflect the concerns and speculations surrounding the motivations and intentions behind the vaccine trial.

“We were told that you came to kill us: ‘they take blood to go and kill people and said that since the whites are smart people, they will reduce our years by using our blood,’ people criticized in one way or another.” (FDG, community health worker, trial participant, woman).

“And one thing doctor, if I may add, you have selected only the people who are not working at the study. Why didn’t you use those who are staff of the trial? That is also a question. As you are there, you are not vaccinated, even the woman, even the man. However, we are vaccinated[…] However, you, the driving forces the trial, you are not vaccinated. Why are you not vaccinated? That is, there is something hidden behind it, or we are sacrificed.” (FGD, medical doctor, trial participant, man).

### Global assessment of the trial.

#### Positive assessment.

The implementation of the EBL2007 vaccine trial was perceived as highly positive in the following respects: 1) the ancillary care policy developed for the management of adverse events not related to the study vaccine mentioned earlier,^23^ 2) the perception of this vaccination as a preparation of the province for a future Ebola epidemic, 3) the renovation of the hospital buildings, 4) provision of water, electricity, and various other equipment to be returned to the general hospital at the end of the trial (offices, cold chain, generators, and satellite antennas for internet, etc.), and 5) capacity building sessions for HCPs and frontline workers prior to each scheduled visit in the trial.^9^

“This study has changed our lives because when the disaster arrived in the Boende health district, precisely in the health area of Lokolia, we saw dead bodies with our own eyes, we lost our brothers, our mothers, our fathers, our children, and when the study arrived, we were satisfied with the arrival of this study because we believed that after the study, it is the solution that will come, maybe soon, we will be more attacked by the disease.” (FGD, nurse head of health center, non-trial participant, man).

“Yes, because first here we had a problem of water supply; with water that we have at the EBOVAC, we often see people asking, ‘may I draw water?’ ‘Yes, you can’[…] Furthermore, we have power supplied 24h/24, that is very good. Our premises are arranged, it was not like that. They are really very good researchers.” (FGD, nurse, trial participant, man).

“[…] Well, since I left the university, I have not yet manipulated the automaton. Through this study, there was the chance to handle this device.” (Interview, nurse, staff in the trial, woman).

Some respondents indirectly mentioned the impact of the vaccine trial process on their adoption of positive behaviors in terms of EVD prevention.

“For instance, we were used to eating bush meat that we had picked up in the forest, but through the study we understood what to eat and what not to eat […] Since then, I have been giving the EBOVAC 4 out of 5.” (FGD, nurse trial participant, woman).

“This study has made me aware of how I can protect myself. The vaccine may also protect me, but I now know how to protect myself from Ebola.” (FGD, first aid worker, trial participant, woman).

#### Negative assessment.

Most interviewees and FGD participants expressed their dissatisfaction with the amount of compensation provided to trial participants in terms of remuneration for their time and reimbursement of travel expenses. Several individuals referred to higher payments made in other Ebola response projects in the country.

“I worked in the control of the Ebola epidemic in the past and I was able to buy a land. Now I am in this study EBOVAC, I only got money for transportation and that’s all.” (FGD, nurse trial participant, woman).

“We noticed that our payment was insufficient. We, as staff. But as it was decided by the chiefs and there was no way to discuss, we started the work because we have to work first, and the payment comes afterwards.” (Interview, trial staff, man).

“We are involved in a study at risk where the drug could cure or potentially cause harm. We who are still alive completed the 3-year study. Did we receive compensation for the risk we took by participating in this vaccine trial?…” (FGD, community health worker, trial participant, man).

Some few others specified that they were partially satisfied with the trial because they were expecting to receive gifts at the end as a sign of reward for their volunteering efforts or some increase in travel expense refunds.

“We are satisfied, but not completely, because we were expecting to receive gifts. We have been involved for 2 years, so we were expecting some gifts.” (FGD, nurse, trial participant, woman).

Let it be noted that because of the scarcity of qualified personnel with experience in conducting clinical trials in a remote area like Boende health district, the recruitment process for local trial staff performed by the principal investigator in collaboration with the chief of the provincial health division of Tshuapa relied primarily on local HCPs who had previously worked in the monkeypox vaccine clinical trial^24^ conducted in the same area. However, some interviewees pointed out that the recruitment process for trial staff was flawed, as the true experts in the field of Ebola response were not hired. They perceived the recruitment as a mere illusion, with the researchers being influenced by local authorities in carrying out the selection process.

**“**I didn’t like the organization because there are specialists in Boende regarding Ebola. I am there, but we were left out. On the contrary, you took the people who did not know Ebola! Normally you should have come to us, to look for – at the provincial Division of Health to look for who are experts, who have already lived, who have already handled Ebola, but you did not do that. You recruited according to you, but the experts, you abandoned us.” (FGD, medical doctor, trialmparticipant, man).

### Perspective on future Ebola virus/Ebola vaccine research.

#### Study site.

Interviewees advocated that the trial site should become the site of future clinical trials and that establishing a well-equipped laboratory to analyze all study samples on-site could overcome beliefs/speculations that arose from shipping collected blood overseas. However, a primary concern expressed was that local authorities may lack the capacity to sustain the project equipment acquired, especially once they were no longer used in another research project.

“We would like to have a study site here and a laboratory to avoid that each time there is a study, we always have to go to a foreign laboratory, given that we are in Boende where there is an epidemic area.” (FGD, community health worker, trial participant, man).

“I believe that this site should not remain unused, otherwise it will be ruined. It will be destroyed. Did you find it like this? There are NGOs that come here and do not set up anything special, they make their tent and then they leave. I tell you; I am now 60 years old, and I have never seen anything like this. I worked with MSF, at the time we were paid 150$, but it was not like that; the whole community is astonished by what happened at EBOVAC, they want this work to continue, especially when the population passes by here, they are astonished by the place, wondering if it was even a hotel.” (FGD, health facility cleaner, trial participant, woman).

### Perspective on Ebola vaccine research.

A significant number of interviewees suggested that the community should be involved in the study protocol design to consider the relevance of the populations to be involved in the trial. For example, they would like to see more projects determining the Ebola reservoir in the forest. Including all professional categories of the population would be a better plan for future EVD preparedness and would enhance Ebola vaccine confidence.

“When a disease arises, it does not affect the civil servants or those who work in the health sector, but the disease affects the whole community. I recommend that when you arrive, you take this initiative so that it is not limited to this place, but that it is extended to other places, and secondly, we will receive it in the same way that you have promised us.” (Interview, trial participant who withdrew consent, man).

## DISCUSSION

This study assessed the experiences of HCPs and frontline workers enrolled and not enrolled in the trial as well as the experiences of the EBL2007 vaccine trial staff and local health authority of the Tshuapa province.

Our findings unveiled positive experiences associated with partaking in the trial, including commitment to improving the trial site and equipping the volunteers with necessary skills and knowledge through frequent workshops. For example, on EVD, particular emphasis was placed on the universal standards of hygiene and sanitation during workshops, as well as good practices for infection control and prevention. Information related to risky behaviors was also reiterated, such as the risk associated with bushmeat consumption or raw meat prepared from unknown animals and the handling of blood and bodily fluids. This might have contributed to a positive trial experience. Additionally, a comprehensive policy of ancillary care support was implemented during the trial, ensuring that participants received the necessary health care and assistance throughout the trial.^23^ This provision of medical support further enhanced the overall experience of interviewees and FGD participants.

Furthermore, our findings show a broad acceptability of the study vaccine and the study site and a tremendous willingness to support the development of Ebola immunization/research among the larger community as part of the DRC preparedness plan for likely future EVD epidemics in areas where Ebola is endemic. Other research exploring the experiences and perception among HCPs and frontline workers participating in Ebola vaccine trials reported similar findings.^22^^,^^25^ This widespread acceptance of an experimental Ebola vaccine and positive experience of the trial may have a positive influence on the general population in the event a vaccination program is established, as the larger population generally relies on local HCPs to seek information regarding any new health innovation.^22^^,^^26^^,^^27^

The objectives of the EBL2007 vaccine trial still appeared to be understood by most respondents 2 years after inclusion. This was made possible by the investigators’ repetition of the study objective prior to each scheduled visit during organized workshops. However, two different perspectives emerged regarding how the interviewees and FGD participants perceived the achievement of the assigned objectives in the trial. From the trial staff’s perspective, a sense of relief and pride was felt as the initial trial assumptions were met with a high retention rate. From the trial participants’ perspective, accepting the risk of receiving an experimental vaccine should be absolutely rewarded (even symbolically) by the research team. According to certain participants, a manifestation of trial participants’ appreciation should be demonstrated by the research team through the provision of items such as bicycles, T-shirts, or other tangible goods. Other interviewees/FGD participants voiced that the disclosure of all the results from the blood samples collected during the trial would suffice, along with results of the experimental vaccine. To comply with this request, the research team plans to organize a conference in Boende to disseminate the main findings of the study once they are available, and all the trial participants will be invited. However, results were not yet available at the time of these interviews and FGDs.

Furthermore, a considerable number of participants perceived the time offered and travel cost refund provided during the trial visits as insufficient. Several authors have described the pursuit of an immediate financial gain beyond the benefit of improving one’s health as an inducement to volunteer in a clinical trial in developing countries, as well as throughout the world, regardless of social or educational status.^28^^–^^30^ However, some studies indicated that excessive compensation is also ethically questionable and can be considered as excessive inducement to participate in a trial.^31^^–^^33^ Yet, studies examining the experiences of HCPs volunteering in Ebola vaccine trials elsewhere reported different results. In fact, the primary motivation to receive an experimental vaccine ahead of the general population has been identified as a strong desire to contribute to the search for an Ebola vaccine.^22^^,^^34^

Most trial participants had concerns regarding blood collection. Blood was perceived to be either bought or stolen by the researchers through blood collection. The shipment of collected blood samples from the trial location to other countries reinforced this belief, leading to rumors among study participants’ social circle accusing the researchers of exchanging the participants’ blood for the money provided for their travel costs and time offered. The refusal to continue in a clinical trial due to religious beliefs prohibiting the collection of human blood has also been reported in previous studies.^35^^,^^36^ Likewise, many people discontinued in studies conducted elsewhere in Africa because they were afraid of getting their blood drawn.^37^^,^^38^ Reluctance towards blood collection in clinical trials may be due to beliefs that blood collected or donated from someone who is not sick is often used for mystical rituals.^38^ Some African cultures often have strong spiritual beliefs and traditional healing practices. Some individuals may believe that blood holds a substantial spiritual or life force, and therefore withdrawing blood could be seen as potentially harmful or weakening. Hence, the use of blood samples in trials conducted in Africa should always be given special attention, and investigators’ efforts should focus on providing consistent and clear risk communication on study procedures during the informed consent process.

Trial staff did not receive the vaccine under investigation to prevent any interference with the evaluation of reactogenicity outcomes among vaccinated participants (interpretation bias). However, this was perceived by some interviewees and FGD participants as indicating that there might be adverse long-term outcomes from the study vaccine, such as death or reduced life expectancy.

This study highlights the complexity of research in resource-limited settings where researchers and trial volunteers are seemingly living in two different worlds. This may suggest that the information provided by the researchers to the trial volunteers prior to signing a consent form would not significantly contribute to adequately enlightening them about all the procedures and planning in accordance with the defined research protocol on the researchers’ side.^39^ Hence, the active commitment of the community during the preparatory phase of research, going beyond mere participation, has increasingly become a crucial and beneficial factor, specifically in clinical trials.^27^^–^^30^ An early partnership between researchers and the community could provide an opportunity for researchers and the community to work together, sharing knowledge and responsibility from the beginning until the end of the project, to create, revise, and generate research knowledge.^40^ In the context of the EBL2007 vaccine trial, early involvement of the conceptual phase of community (the protocol) would have created opportunities to consider real-life scenarios. In doing so, problems related to the volunteers’ expectations regarding the reimbursement of time offered and transport, the duration, and the number of reasonable blood samples could have been discussed before the trial started recruitment.

### Limitations and strengths.

First, our study included some HCPs and frontline workers who had experienced an Ebola outbreak in the past. The perception of disease risk among interviewees/FGD participants who had faced the disease could have affected our findings, and thus these findings might not be applicable to those who have not faced a previous Ebola outbreak. Second, the involvement of one of the qualitative research teams in the EBL2007 vaccine trial as a subinvestigator during conversations may have affected our findings. Finally, conducting interviews in locations close to the study site could influence the views of some participants and potentially impact our results. Some of the selected conversation locations were situated within the Boende HGR premises, which served as the EBL2007 vaccine trial site. This choice of location might have created a sense of unease among participants regarding the expression of opinions or views that could potentially upset or offend the researchers. The fear of potential reprisals from their hierarchical superiors at the hospital could have contributed to this apprehension.

However, the inclusion of different job professions and sexes in the FGDs and individual interviews allowed us to gather a detailed understanding of the participants’ experiences in the clinical trial and their overall acceptance of the vaccine being studied as well as those of nonparticipants, trial staff, and members of local health authorities. This approach helped us capture a more comprehensive picture of the trial experience among the individuals we investigated.

## CONCLUSION

Interviewees and FGD participants voiced positive experiences gained from volunteering in the trial. These included Ebola prevention training, Ebola vaccination, ancillary care provided by researchers, and hospital renovations at the trial site. Furthermore, a widespread acceptance of the study vaccine and the trial site, as well as a strong willingness to support the development of Ebola vaccination and research in the wider community as part of an EVD preparedness plan, was reported.

Areas of uncertainty and ambiguity pertaining to the understanding of compensation costs, as well as concerns regarding blood collection and its exportation to other countries, were raised. By involving the community of the study area in which the research will take place already in the study design, researchers can introduce acceptable trial procedures before the start of recruitment or anticipate any concerns. In addition, it can enable a better community comprehension of the reasons why a certain population is chosen to participate in a trial and the challenges that they may face.

These elements combined can add to fair expectations of both trial participants and community members, which in turn can contribute to their trust in the research.

## Supplemental Materials

10.4269/ajtmh.23-0581 Supplemental Materials

## Footnotes

^1^
A sponsor staff member of the research project (EBL2007 vaccine trial).

^2^
Lokolia is the health area where the index case of the 2014 epidemic occurring in the Boende health district was observed.
