# Supplementary material for: Healthcare Providers’ and Frontline Workers’ Experiences of an Ebola Vaccine Trial in the Boende Health District of the Democratic Republic of the Congo
Source: Am J Trop Med Hyg. 2024 Jul 2;111(3):578–88. doi: 10.4269/ajtmh.23-0581 (PMC11376161; doi:10.4269/ajtmh.23-0581)
Supplement: Supplemental Materials [file tpmd230581.SD1.pdf]

Appendix 1

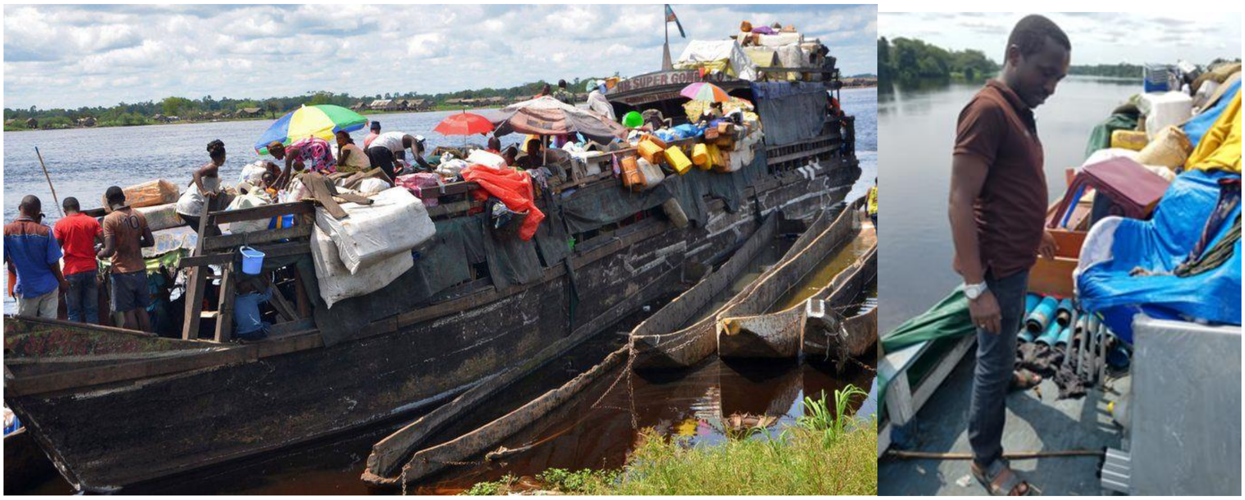

Figure 1. A boat propelled by a whale-shaped engine that transports people from Boende to Kinshasa, passing through Mbandaka (Equateur province)

Appendix 2

Table 1: Overview of Ebola outbreaks in the DRC. Source: CDC (https://www.cdc.gov/vhf/ebola/history/chronology.html, accessed 27 September 2022)

Outbreak	Year	Place	No of fatalities	No of cases	Before or after start of EBL2007
**1**	1976	Yambuku, Equateur (Mongala)	280	318	Before
**2**	1977	Tandala, Equateur (Sud Ubangi)	1	1	Before
**3**	1995	Kikwit, Kwilu	254	315	Before
**4**	2007	Mweka, Kasai	187	264	Before
**5**	2008	Mweka, Kasai	15	32	Before
**6**	2012	Isiro, Haut Uelé	13	38	Before
**7**	2014	Boende, Equateur (Tshuapa)	49	69	Before
**8**	2017	Likati, Bas Uelé	4	8	Before
**9**	2018	Mbandaka, Equateur	33	54	Before
**10**	2018-2020	North Kivu	2287	3470	Before; After*
**11**	2020	Mbandaka, Equateur	55	130	After
**12**	2021 (Feb-May)	North Kivu	6	12	After
**13**	2021 (Oct-Dec)	North Kivu	6	11	After
**14**	2022	Mbandaka, Equateur	5	5	After
**15**	2022	North Kivu	1	1	After

***** The EBL2007 trial started on the 18th of December 2019. The North Kivu Ebola epidemic started in July 2018, before the start of the EBL2007 trial but lasted until June 2020, which if after the start of the trial.

Supplement 1. Thematic guide for focus groups (FGDs) with trial volunteers

Theme 1: Understanding the clinical trial in general and the protocol of the EBL2007 trial in Boende

1.Can you describe your understanding of the EBL2007 clinical trial's objective??

- In your opinion,. "Do you feel the objective of the trial was achieved? Could you elaborate?"

2. What did you consider in agreeing to be enrolled in the EBL2007 study (vaccine trial)?

3. Could you share any concerns or apprehensions you had when enrolling in the study?

4. What were your expectations of the EBL2007 study?

- Have these expectations and hopes been fulfilled? Did the clinical trial meet these expectations and hopes? If yes, how? If not, why not?

5. How do you like the organization of the study? What were the most and least clear aspects during the trial? Did you encounter any difficulties or difficult moments during the trial? Which ones?

- Were the staff competent to conduct this study? Can you give us an example that shows us that?
- Were the means and resources involved in conducting such a study in an environment like Boende well measured?

6. Should the study be conducted elsewhere where there is a greater need for people to be vaccinated? Where, for example? What are the reasons why this study should be conducted elsewhere instead? Who should be vaccinated in this study?

7. Why do you think it was important to conduct this clinical trial in Boende?

8.? How has participating in this study impacted your daily life? Have there been any changes in your behavior as a result?

9. What impact do you think this study has had on the broader community not involved in it?"

10. How can the community that was not part of this study benefit from the impact of this study?

- (if respondents answer: "through sensitization") How should sensitization be done? Who should deliver the message to community members?

11. What role, if any, do you think politics played in the development of this trial?

12. What were the social facts and events around you that had an impact on the progress of the trial?

13. How did the standard of living (economic) in Boende affect the conduct of the trial? Did being rich or poor have an impact on the conduct of the study?

Theme 2: Looking to the Future

14. How did you experience the last appointment of the trial? What were the most and least clear aspects?

15. How do you see the future of the trial site? What are your expectations and hopes for it?

16. After your participation in this trial, how do you view the study that took place and the Ebola vaccine research? What are your expectations and hopes? What aspects should be improved, and what are your suggestions and recommendations?

Supplement 2. Thematic Guide for Focus Group Discussions (FGDs) with Study Staff

Topic 1: Experience with the clinical trial in general and the EBL2007 protocol in Boende

1. Can you describe your role in the study and any changes you experienced during the study period?

2. How would you describe the purpose of the EBL2007 study?

- Do you feel that the study achieved its purpose? Could you elaborate?".

3. What concerns or apprehensions did you have at the beginning of the study?

4. How would you assess the organization of the study, including both its clear and unclear aspects? Did you face any challenges or significant moments during the study? How did you manage them?"

5. Can you share your thoughts on your psychological well-being throughout the study?

6. How was your communication and collaboration with the rest of the medical staff? What difficulties did you encounter and how did you overcome them?

7. How was your communication and collaboration with other stakeholders in the EBOVAC project? What difficulties did you encounter and how did you overcome them?

8. Do you think the resources and means utilized for conducting the study in an environment like Boende were appropriate?

9. Should the study be conducted elsewhere where there is a greater need for people to be vaccinated? Where, for example? What are the reasons why this study should be conducted elsewhere instead? Who should be vaccinated in this study?

10. What are the reasons why this clinical trial had to take place here in Boende this study?

11. How has this study influenced your daily life: what behaviors have you changed because of participating in this study?

12. How has this study influenced the participants' daily lives? From what you can observe, what behaviors did they change because of participating in this study?

13. Were there any relationships with the community? Can you describe them for us?

14. In your estimation, what is the contribution of this study on the broader community that was not part of this study?

15. How can the community that was not part of this study benefit from the impact of this study?

a. (if respondents answer: "through sensitization") How should sensitization be done? Who should deliver the message to community members?

16. What role, if any, do you believe politics played in the development of this trial?

17. What were the social facts and events around you that had an impact on the progress of the trial?

18. How did the standard of living (economic) in Boende affect the conduct of the trial? Did being rich or poor have an impact on the conduct of the study?

Theme 2: Looking to the Future

19. Given the capacity building received during the study, how do you see your career trajectory after the trial ends?

20. How do you see the future of the study site: what are your expectations and hopes for it?

21. After your participation in this trial, how do you see Ebola vaccine research? What are your expectations and hopes? What aspects should be improved, and what are your suggestions and recommendations?

Supplement 3. Interview guide for individual in-depth interviews (IIIs) with volunteers who withdrew their consent to the study

Theme 1: Understanding the clinical trial in general and the protocol of the EBL2007 trial in Boende

1. How would you describe the purpose of the EBL2007 study? Do you feel that the study achieved its purpose? Could you elaborate?"

2. What did you consider in agreeing to be enrolled in the EBL2007 study?

3. What concerns or apprehensions did you have at the beginning of the study?

4. What did you expect the EBL2007 study to provide?

- Have these expectations and hopes been fulfilled? Did the clinical trial meet these expectations and hopes? If yes, how? If not, why not?

5. What led you to withdraw consent from this study?

6. During your participation in the study, how do you appreciate the organization of the study: what were the most and least clear aspects? Did you encounter any difficulties or difficult moments during the trial? Which ones?

- Were the staff competent to conduct this study? Can you give us an example that shows us that?
- Were the means and resources involved in conducting such a study in an environment like Boende well measured?

7. Should the study be conducted elsewhere where there is a greater need for people to be vaccinated? Where, for example? What are the reasons why this study should be conducted elsewhere instead? Who should be vaccinated in this study?

8. What are the reasons why this clinical trial had to take place here in Boende this study?

9. How has this study influenced your daily life: what behaviors have you changed because of participating in this study?

10. How has this study influenced the participants' daily lives? From what you can observe, what behaviors did they change because of participating in this study?

11. What is the contribution of this study on the larger community that was not part of this study?

12. How can the community that was not part of this study benefit from the impact of this study?

a. (if respondents answer: "through sensitization") How should sensitization be done? Who should deliver the message to community members?

13. What was the role of politics in the development of this trial?

14. What were the social facts and events around you that had an impact on the progress of the trial?

15. How did the standard of living (economic) in Boende affect the conduct of the trial? Did being rich or poor have an impact on the conduct of the study?

Theme 2: Looking to the Future

16. After you withdrew your consent, do you still hold your position or would you have changed your mind? Why or why not?

17. How do you see the future of the study site: what are your expectations and hopes for it?

18. After your participation in this trial, how do you see Ebola vaccine research? What are your expectations and hopes? What aspects should be improved, and what are your suggestions and recommendations?

Supplement 4. Interview Guide for In-depth Individual Interviews (III) with Health Authorities

Topic 1: Experience with the clinical trial in general and the EBL2007 protocol in Boende

1. What do you think was the purpose of the study?

- In your opinion, has this objective been achieved? Explain.

2. What did you expect the EBL2007 study to bring to Boende?

- Were these expectations and hopes fulfilled. Did the study meet these expectations and hopes? If so, how? If not, why not?

3. How do you rate the organization of the study: what were the most and least clear aspects of the trial?

- Were the staff competent to conduct this study? Can you give us an example that shows us that?
- Were the means and resources involved in conducting such a study in an environment like Boende well measured?

4. How was your communication and collaboration with the EBOVAC project staff? What difficulties did you encounter and how did you overcome them?

5. Should the study be conducted elsewhere where there is a greater need for people to be vaccinated? Where, for example? What are the reasons why this study should be conducted elsewhere instead? Who should be vaccinated in this study?

6. What are the reasons why this study had to take place here in Boende?

7. Has this study had an impact on your daily life? What impact has it had? What behaviors have you changed because of your involvement in this study?

8. How has this study influenced the participants' daily lives? From what you can observe, what behaviors did they change because of participating in this study?

9. Were there any relationships between the EBOVAC project and the community? Can you describe them for us?

10. In your estimation, what is the contribution of this study on the broader community that was not part of this study?

11. How can the community that was not part of this study benefit from the impact of this study?

a. (if respondents answer: "through sensitization") How should sensitization be done? Who should deliver the message to community members?

12. What was the relationship between politics and the conduct of this trial?

13. What were the social facts and events around you that had an impact on the progress of the trial?

14. How did the standard of living (economic) in Boende affect the conduct of the trial? Did being rich or poor have an impact on the conduct of the study?

Theme 2: Looking to the Future

15. Given the capacity building received by health professionals during the study, how do you see their career trajectories after the study ends?

16. How do you see the future of the study site: what are your expectations and hopes for it?

17. After your participation in this trial, how do you see Ebola vaccine research? What are your expectations and hopes? What aspects should be improved, and what are your suggestions and recommendations?

Appendix 5. 5.1.1.1_Appendix V: Thematic Guide for Focus Group Discussions (FGDs) and Individual In-depth Interviews (IIIs) with Community Members

Theme 1: Understanding the clinical trial in general and the protocol of the EBL2007 trial in Boende

1. Have you been aware of an Ebola vaccine study going on here in Boende? If so, since when?

2. What do you think was the purpose of this study?

- In your opinion, has this objective been achieved? Explain.

3. What did you expect this study to bring to your community?

- Did this study meet these expectations and hopes? If so, how? If not, why not?

4. What were your fears when you learned that an Ebola vaccination study was going to take place here in Boende?

5. What do you think were the reasons why participants enrolled in the vaccine trial? What benefits did they expect, and what benefits did they get?

6. How do you feel about the organization of the study: what were the most and least clear aspects during the trial? Did you observe any difficulties or difficult moments during the trial? Which ones?

- Do you think the staff was competent to conduct this study? Why or why not?
- Do you think that the means and resources involved were well measured to conduct this study in an environment like Boende?

7. Do you think it was a good decision to conduct this study in Boende, or should the study have been conducted elsewhere? Why or why not? If elsewhere, where?

8. Who do you think should receive this vaccine first in this study? Do you think it was a good decision to vaccinate health care workers, or who should be vaccinated in this study?

9. How was your relationship with the study staff (EBOVAC project staff)? Was there any communication? Did you encounter any difficulties? How did you overcome them?

10. Has this study had an impact on your daily life? What behaviors have you changed because of this study?

11. If there was no impact, how could the community that was not part of this study benefit from this study?

12. Did you observe any events, at the policy level, that influenced the course of this trial?

13. Did you observe any societal facts or events that happened around you that had an impact on how the trial went?

14. How did the standard of living (economic) in Boende affect the conduct of the trial? Did being rich or poor have an impact on the conduct of the study?

Theme 2: Looking to the Future

15. How do you see the future of the trial site at RGH Boende: what are your expectations and hopes for it?

16. How do you see the future of Ebola vaccine research: what are your expectations and hopes? What aspects should be improved, and what are your suggestions and recommendations?
